# Effects of Sofosbuvir/Velpatasvir Therapy on Extrahepatic Manifestations in Patients With Type 2 Diabetes and Chronic Hepatitis C: Insights From a Nationwide Hepatitis C Virus Registry in Taiwan

**DOI:** 10.1002/kjm2.70067

**Published:** 2025-06-20

**Authors:** Szu‐Jen Wang, Te‐Sheng Chang, Ching‐Chu Lo, Chao‐Hung Hung, Chien‐Wei Huang, Lee‐Won Chong, Pin‐Nan Cheng, Ming‐Jong Bair, Ming‐Lun Yeh, Cheng‐Yuan Peng, Chien‐Yu Cheng, Jee‐Fu Huang, Chih‐Lang Lin, Chi‐Chieh Yang, Hsing‐Tao Kuo, Tsai‐Yuan Hsieh, Tzong‐Hsi Lee, Pei‐Lun Lee, Wen‐Chih Wu, Chih‐Lin Lin, Wei‐Wen Su, Sheng‐Shun Yang, Chia‐Chi Wang, Jui‐Ting Hu, Lein‐Ray Mo, Chun‐Ting Chen, Yi‐Hsiang Huang, Chun‐Chao Chang, Chia‐Sheng Huang, Guei‐Ying Chen, Chien‐Neng Kao, Chi‐Ming Tai, Chun‐Jen Liu, Mei‐Hsuan Lee, Pei‐Chien Tsai, Shu‐Chi Wang, Chia‐Yen Dai, Jia‐Horng Kao, Han‐Chieh Lin, Wang‐Long Chuang, Kuo‐Chih Tseng, Chi‐Yi Chen, Chung‐Feng Huang, Ming‐Lung Yu

**Affiliations:** ^1^ Graduate Institute of Clinical Medicine Kaohsiung Medical University Kaohsiung Taiwan; ^2^ Division of Hepatogastroenterology, Department of Internal Medicine Shin Huey Shin Hospital Kaohsiung Taiwan; ^3^ Division of Gastroenterology, Department of Internal Medicine Yuan's General Hospital Kaohsiung Taiwan; ^4^ Division of Hepatogastroenterology, Department of Internal Medicine ChiaYi Chang Gung Memorial Hospital Chiayi Taiwan; ^5^ College of Medicine Chang Gung University Taoyuan Taiwan; ^6^ Division of Gastroenterology, Department of Internal Medicine St. Martin De Porres Hospital Chiayi Taiwan; ^7^ Division of Hepatogastroenterology, Department of Internal Medicine Kaohsiung Chang Gung Memorial Hospital Kaohsiung Taiwan; ^8^ Division of Gastroenterology Kaohsiung Armed Forces General Hospital Kaohsiung Taiwan; ^9^ Division of Hepatology and Gastroenterology, Department of Internal Medicine Shin Kong Wu Ho‐Su Memorial Hospital Taipei Taiwan; ^10^ School of Medicine Fu‐Jen Catholic University New Taipei City Taiwan; ^11^ Division of Gastroenterology and Hepatology, Department of Internal Medicine National Cheng Kung University Hospital, College of Medicine, National Cheng Kung University Tainan Taiwan; ^12^ Division of Gastroenterology, Department of Internal Medicine Taitung Mackay Memorial Hospital Taitung Taiwan; ^13^ Mackay Medical College New Taipei City Taiwan; ^14^ Hepatobiliary Division, Department of Internal Medicine Kaohsiung Medical University Hospital, Kaohsiung Medical University Kaohsiung Taiwan; ^15^ Hepatitis Research Center, College of Medicine; Center for Metabolic Disorders and Obesity Kaohsiung Medical University Kaohsiung Taiwan; ^16^ Center for Digestive Medicine, Department of Internal Medicine China Medical University Hospital Taichung Taiwan; ^17^ School of Medicine China Medical University Taichung Taiwan; ^18^ Division of Infectious Diseases, Department of Internal Medicine, Taoyuan General Hsopital Ministry of Health and Welfare Taoyuan Taiwan; ^19^ Liver Research Unit, Department of Hepato Gastroenterology and Community Medicine Research Center Chang Gung Memorial Hospital at Keelung, College of Medicine, Chang Gung University Keelung Taiwan; ^20^ Department of Gastroenterology, Division of Internal Medicine Show Chwan Memorial Hospital Changhua Taiwan; ^21^ Division of Gastroenterology and Hepatology, Department of Internal Medicine Chi Mei Medical Center Tainan Taiwan; ^22^ Division of Gastroenterology, Department of Internal Medicine Tri Service General Hospital, National Defense Medical Center Taipei Taiwan; ^23^ Division of Gastroenterology and Hepatology Far Eastern Memorial Hospital New Taipei City Taiwan; ^24^ Division of Gastroenterology and Hepatology, Department of Internal Medicine Chi Mei Medical Center, Liouying Tainan Taiwan; ^25^ Wen‐Chih Wu Clinic, Fengshan Kaohsiung Taiwan; ^26^ Department of Gastroenterology Renai Branch, Taipei City Hospital Taipei Taiwan; ^27^ Department of Gastroenterology and Hepatology Changhua Christian Hospital Changhua Taiwan; ^28^ Division of Gastroenterology and Hepatology, Department of Internal Medicine Taichung Veterans General Hospital Taichung Taiwan; ^29^ Taipei Tzu Chi Hospital, Buddhist Tzu Chi Medical Foundation and School of Medicine Tzu Chi University Taipei Taiwan; ^30^ Liver Center, Cathay General Hospital Taipei Taiwan; ^31^ Division of Gastroenterology Tainan Municipal Hospital (Managed By Show Chwan Medical Care Corporation) Tainan Taiwan; ^32^ Division of Gastroenterology, Department of Internal Medicine tri Service General Hospital Penghu Branch, National Defense Medical Center Taipei Taiwan; ^33^ Department of Medical Research Taipei Veterans General Hospital Taipei Taiwan; ^34^ Institute of Clinical Medicine, College of Medicine National Yang Ming Chiao Tung University Taipei Taiwan; ^35^ Division of Gastroenterology and Hepatology, Department of Internal Medicine Taipei Medical University Hospital Taipei Taiwan; ^36^ Division of Gastroenterology and Hepatology, Department of Internal Medicine, School of Medicine, College of Medicine Taipei Medical University Taipei Taiwan; ^37^ Yang Ming Hospital Chiayi Taiwan; ^38^ Penghu Hospital, Ministry of Health and Welfare Penghu Taiwan; ^39^ National Taiwan University Hospital Hsin‐Chu Branch Hsinchu Taiwan; ^40^ Division of Gastroenterology and Hepatology, Department of Internal Medicine E‐Da Hospital, I‐Shou University Kaohsiung Taiwan; ^41^ School of Medicine for International Students, College of Medicine I‐Shou University Kaohsiung Taiwan; ^42^ Hepatitis Research Center and Department of Internal Medicine National Taiwan University Hospital Taipei Taiwan; ^43^ Department of Medical Laboratory Science and Biotechnology Kaohsiung Medical University Kaohsiung Taiwan; ^44^ School of Medicine Tzuchi University Hualien Taiwan; ^45^ Department of Internal Medicine Dalin Tzu Chi Hospital, Buddhist Tzu Chi Medical Foundation Chiayi Taiwan; ^46^ Division of Gastroenterology and Hepatology, Department of Medicine Ditmanson Medical Foundation Chiayi Christian Hospital Chiayi Taiwan; ^47^ Ph.D. Program in Translational Medicine, College of Medicine Kaohsiung Medical University, Academia Sinica Kaohsiung Taiwan; ^48^ Center for Liquid Biopsy and Cohort Research Kaohsiung Medical University Kaohsiung Taiwan

**Keywords:** chronic hepatitis C, HbA1c, real‐world, Taiwan, type 2 diabetes mellitus

## Abstract

This study examines the impact of hepatitis C virus (HCV) eradication through sofosbuvir/velpatasvir (SOF/VEL) treatment on glycated hemoglobin (HbA1c) levels in patients with chronic hepatitis C and type 2 diabetes mellitus (T2DM). Utilizing data from the Taiwan HCV Registry, a retrospective analysis was conducted on 2180 patients who met the inclusion criteria, 695 of whom had T2DM. HbA1c levels significantly decreased in the diabetes group from 7.32% ± 1.72% at baseline to 6.87% ± 1.34% after achieving sustained virological response (SVR12). Patients with higher baseline HbA1c levels and cirrhosis experienced more pronounced HbA1c reductions. Among diabetic patients with HbA1c levels ≥ 6.5, 24.6% achieved levels < 6.5 following HCV elimination, while 24.4% of prediabetic patients observed HbA1c reductions < 5.7. Multivariate analysis identified fasting glucose levels and diabetes status as significant factors associated with HbA1c decline. These findings suggest that successful HCV treatment can improve glycemic control, highlighting the need for collaboration between hepatology and non‐hepatology specialists in patient care.

## 1 Introduction

The hepatitis C virus (HCV) was identified in 1989 and is a major global health concern, with an estimated 56.8 million infections (0.7% prevalence) worldwide as of January 1, 2020 [1]. The World Health Organization aims to control HCV by 2030 by promoting decentralized testing, task shifting, and simplified direct‐acting antiviral (DAA) therapies at the primary care level [2, 3].

Patients with chronic hepatitis C (CHC) are at risk of liver complications and metabolic dysfunction, such as prediabetes and diabetes [4, 5]. Notably, type 2 diabetes mellitus (T2DM) is an extrahepatic manifestation of CHC. HCV eradication through antiviral therapy reduces hepatic complications and leads to improved metabolic outcomes [6, 7, 8], potentially reducing insulin resistance, enhancing beta‐cell function [9, 10], and reducing the incidence of diabetes [8]. However, improvements in glycemic control may be transient rather than long‐lasting [11, 12].

Sofosbuvir/velpatasvir (SOF/VEL), a 12‐week protease inhibitor‐free regimen for CHC, has achieved sustained virologic response (SVR) rates of 95%–100% in over 1100 clinical trial patients across HCV genotypes 1–6 [13]. The efficacy of SOF/VEL has also been validated in real‐world settings through multiple studies in Western [14] and Asian populations [15].

Glycated hemoglobin (HbA1c) levels are a highly sensitive and specific indicator used in diabetes screening [16, 17]. Studies have demonstrated that HbA1c levels are typically reduced after HCV eradication [18, 19]; however, the effects of HCV eradication with SOF/VEL on glycemic control and changes in HbA1c levels have not been extensively examined in nationwide studies. The current study filled this gap by analyzing pretreatment and posttreatment HbA1c levels in a large multicenter cohort of patients with CHC from the Nationwide HCV Registry Program (TACR).

## 2 Materials and Methods

### 2.1 Patients

This retrospective analysis included adult patients (age ≥ 20) who completed CHC treatment with SOF/VEL between August 2019 and December 2022 whose data were accessible through the TACR registry. Patients were eligible if they had SVR results at a 12‐week post‐SOF/VEL‐treatment follow‐up (SVR12), had HbA1c data at baseline and at a 12‐week post‐treatment follow‐up, did not use ribavirin, and had not experienced DAA treatment. Patients with a history of diabetes mellitus (DM), an HbA1c level > 6.5%, or a fasting glucose level > 126 mg/dL were classified as having DM. Patients for whom SVR12 results or HbA1c data at baseline and SVR12 were missing or who did not achieve SVR12 were excluded. All patients received SOF/VEL treatment in accordance with Taiwan's national insurance reimbursement criteria and regional guidelines [20, 21, 22]. The present study was conducted in accordance with the principles of the Declaration of Helsinki, and written informed consent was obtained from all participants. The ethics committee of Kaohsiung Medical University Hospital approved this study.

### 2.2 Laboratory and Histological Analyses

Biochemical analyses were conducted using a multichannel autoanalyzer (Hitachi Inc., Tokyo, Japan). HCV antibody levels were measured with a third‐generation enzyme immunoassay (Abbott Laboratories, North Chicago, IL, USA). Additionally, HCV RNA was tested using real‐time polymerase chain reaction (Abbott Molecular, Des Plaines IL, USA, or Roche Diagnostics, Branchburg, NJ, USA) with a detection limit of 15 IU/mL. HCV genotypes were determined using assays from Roche Diagnostics (Roche Molecular Diagnostics, California, US) or Abbott Molecular (Abbott Molecular, Illinois, US), depending on the individual participating site. SVR was defined as undetectable HCV RNA (< 12 IU/mL) at 12 weeks after treatment. The fibrosis‐4 (FIB‐4) index value was calculated using the formula: {age (years) × aspartate aminotransferase (AST, units/l)}/{(platelets109/L) × (alanine transaminase [ALT, units/l])1/2}. Liver cirrhosis was diagnosed through histology, transient elastography, or evidence of portal hypertension or cirrhosis. Finally, fatty liver was assessed by trained physicians using ultrasonography. HbA1c levels were measured at baseline and at 12 weeks after treatment.

### 2.3 Statistical Analysis

Statistical analyses were conducted using SPSS 25 (IBM, Armonk, NY, USA). Frequencies were compared using the *χ* ^2^ test with Yates' correction or Fisher's exact test. Group means were compared using an analysis of variance and Student's *t*‐test, and changes in HbA1c levels were analyzed using paired *t*‐tests and McNemar's test. Stepwise logistic regression was employed to identify factors associated with HbA1c reduction, with consideration of covariates with a *p*‐value of < 0.1 in a univariate analysis. All analyses were two‐sided, and a *p*‐value of < 0.05 was considered significant. A significant decrease in HbA1c was defined as the 75th percentile of the change in HbA1c levels before and after treatment within the study population.

## 3 Results

### 3.1 Patient Demographics

**FIGURE 1:**
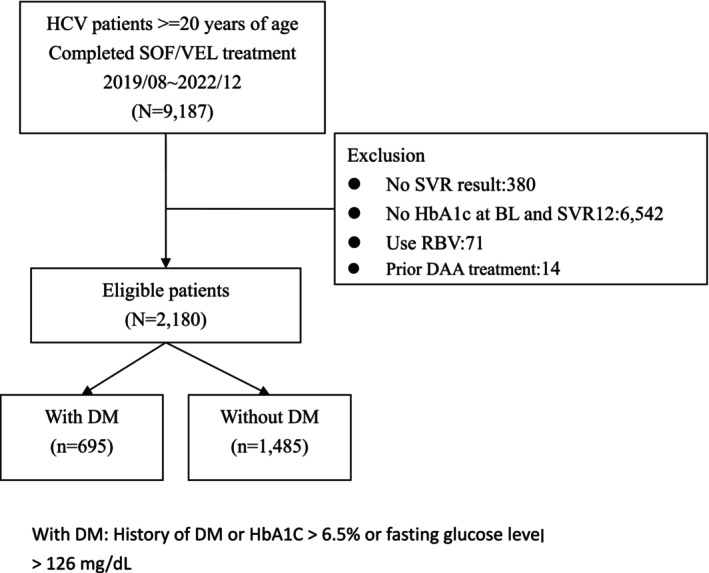
Flow chart of patient enrollment. BL, baseline; DAA, direct‐acting antiviral; DM, diabetes mellitus; HbA1c, glycated hemoglobin; HCV, hepatitis C virus; RBV, ribavirin; SOF/VEL, sofosbuvir/velpatasvir; SVR, sustained viral response; SVR12, undetectable HCV RNA concentration at a 12‐week posttreatment follow‐up.

Data were collected from the TACR platform for the period between August 2019 and December 2022. Among the 9187 patients with CHC who completed SOF/VEL treatment, 380 without SVR results, 6542 without HbA1c data at both baseline and SVR12, 71 on ribavirin treatment, and 14 who had experienced DAA treatment were excluded. Consequently, 2180 patients with available treatment outcomes were included, that is, 695 patients with DM and 1485 without DM (Figure 1).

The mean age and body mass index (BMI) of the cohort were 61.0 years and 24.8 kg/m^2^, respectively. In total, 48.8% of the patients were men (*n* = 1063), and 12% were people who inject drugs (PWID; *n* = 261). Liver cirrhosis was present in 14.4% of the patients (*n* = 314), of whom 1.1% had decompensated cirrhosis (*n* = 23). A total of 94 patients (4.3%) had a history of hepatocellular carcinoma (HCC). The most common viral genotype was GT2 (*n* = 1001, 45.9%), followed by GT1 (*n* = 838, 38.4%). The mean pretreatment HCV RNA level was 5.87 log_10_ IU/mL, and the baseline mean estimated glomerular filtration rate (eGFR) was 88.0 mL/min/1.73m^2^. Most patients (*n* = 2120, 97.3%) were treatment‐naive. The patients' average pretreatment HbA1c level was 6.09%. Among the 2180 patients, 99.2% (*n* = 2162) achieved SVR12 (Table 1).

**TABLE 1:** Baseline patient characteristics.

Characteristics	Total (*n* = 2180)	DM (*n* = 695)	Non‐DM (*n* = 1485)	*p*
Age (years), mean ± SD	61.0 ± 13.0	65.0 ± 11.8	59.1 ± 13.0	< 0.001*
Male, *n* (%)	1063 (48.8)	347 (49.9)	716 (48.2)	0.456
BMI (kg/m^2^), mean ± SD	24.8 ± 2.1	26.1 ± 4.3	24.3 ± 3.9	< 0.001*
Treatment history				
Naïve, *n* (%)	2120 (97.3)	672 (96.7)	1448 (97.5)	0.278
Experienced, *n* (%)	60 (2.8)	23 (3.3)	37 (2.5)	
HBsAg (+), *n* (%)	169 (7.8)	55 (7.9)	114 (7.7)	0.847
HIV co‐infection, *n* (%)	67 (3.1)	7 (1.0)	60 (4.0)	< 0.001*
Smoker, *n* (%)	820 (37.6)	239 (34.4)	581 (39.1)	0.033*
Alcoholism, *n* (%)	590 (27.1)	168 (24.2)	422 (28.4)	0.038*
Hypertension, *n* (%)	883 (40.5)	427 (61.4)	456 (30.7)	< 0.001*
PWID, *n* (%)	261 (12.0)	54 (7.8)	207 (13.9)	< 0.001*
PWID Tx, *n* (%)	101 (4.6)	16 (0.7)	85 (5.7)	< 0.001*
Cirrhosis, *n* (%)	314 (14.4)	133 (19.1)	181 (12.2)	< 0.001*
Decompensated cirrhosis, *n* (%)	23 (1.1)	13 (1.9)	10 (0.7)	< 0.001*
HCC, *n* (%)	94 (4.3)	50 (7.2)	44 (3.0)	< 0.001*
Liver transplantation, *n* (%)	1 (0.1)	1 (0.1)	0 (0.0)	0.144
Dialysis, *n* (%)	47 (2.2)	36 (5.2)	11 (0.7)	< 0.001*
HCV RNA (log_10_ IU/mL), mean ± SD	5.87 ± 1.06	5.98 ± 1.01	5.82 ± 1.08	< 0.001*
HCV genotype 1/2/3/4/5/6/Others, *n* (%)	838 (38.4)/1001 (45.9)/54 (2.5)/1 (0.1)/1 (0.1)/219 (10.1)/66 (3.0)	243 (35.0)/376 (54.1)/14 (2.0)/0 (0.0)/0 (0.0)/43 (6.2)/19 (2.7)	595 (40.1)/625 (42.1)/40 (2.7)/1 (0.1)/1 (0.1)/176 (11.0)/47 (3.2)	< 0.001*
T‐bil (mg/dL), mean ± SD	0.81 ± 0.40	0.76 ± 0.40	0.83 ± 0.40	< 0.001*
AST (IU/L), mean ± SD	57.0 ± 50.1	58.3 ± 49.2	56.4 ± 50.5	0.398
ALT (IU/L), mean ± SD	68.3 ± 73.0	71.0 ± 71.9	67.0 ± 73.5	0.230
Albumin (mg/dL), mean ± SD	4.21 ± 0.43	4.12 ± 0.47	4.24 ± 0.40	< 0.001*
INR, mean ± SD	1.04 ± 0.35	1.05 ± 0.37	1.04 ± 0.34	0.976
Platelets, mean ± SD	197.2 ± 71.8	194.1 ± 75.6	198.6 ± 70.0	0.192
HbA1c (%), mean ± SD	6.09 ± 1.34	7.33 ± 1.72	5.50 ± 0.43	< 0.001*
Creatinine (mg/dL), mean ± SD	1.02 ± 1.08	1.30 ± 1.66	0.89 ± 0.62	< 0.001*
eGFR, mL/min/1.73 m^2^, mean ± SD	88.0 ± 28.9	79.5 ± 32.7	92.0 ± 26.0	< 0.001*
SVR 12 (%)	2162 (99.2)	685 (98.6)	1477 (99.5)	0.030*

Abbreviations: ALT, alanine aminotransferase; AST, aspartate aminotransferase; BMI, body mass index; eGFR, estimated glomerular filtration rate; FIB‐4, fibrosis‐4 index; HbA1c, glycated hemoglobin; HCC, hepatocellular carcinoma; HCV, hepatitis C virus; PWID, people who inject drugs; SD, standard deviation; SVR, sustained viral response; T. bil., total bilirubin.

* *p* < 0.05.

The patients with diabetes were older (65.0 vs. 59.1 years, *p* < 0.001) and had higher BMI (26.1 vs. 24.3 kg/m^2^, *p* < 0.001), serum creatinine levels (1.30 vs. 0.89 mg/dL, *p* < 0.001), and HCV RNA levels (5.98 vs. 5.82 log_10_ IU/mL, *p* < 0.001) as well as lower total bilirubin levels (0.76 vs. 0.83 mg/dL, *p* < 0.001), albumin levels (4.12 vs. 4.24 mg/dL, *p* < 0.001), and eGFRs (79.5 vs. 92.0 mL/min/1.73m^2^, *p* < 0.001) values than those without diabetes did. They also had a higher incidence of hypertension (61.4% vs. 30.7%, *p* < 0.001), liver cirrhosis (19.1% vs. 12.2%, *p* < 0.001), HCC (7.2% vs. 3.0%, *p* < 0.001), and use of dialysis (5.2% vs. 0.7%). The mean pretreatment HbA1c levels were 7.33% in the patients with DM and 5.5% in those without DM (*p* < 0.001; Table 1).

### 3.2 Changes in HbA1c Levels Within Subgroups After DAA Treatment

Among the 2180 patients, 2162 achieved SVR, and 18 did not. In the patients who achieved SVR, HbA1c levels significantly decreased from 6.08% ± 1.34% at baseline to 5.95% ± 1.07% at SVR12 (*p* < 0.001), with a mean reduction of 0.13% ± 0.92%. A significant reduction in posttreatment HbA1c levels was noted across all SVR subgroups, with the exception of those with a history of non‐DM with cirrhosis (5.26% ± 0.47% vs. 5.34% ± 0.52%, *p* = 0.002), those with a history of non‐DM without cirrhosis (5.53% ± 0.41% vs. 5.54% ± 0.51%, *p* = 0.791), and patients with DM with pretreatment HbA1c levels less than the baseline average (5.87% ± 0.45% vs. 6.08% ± 0.99%, *p* = 0.001; Table 2).

**TABLE 2:** Changes in HbA1c levels after SVR by subgroup.

	Baseline HbA1c	SVR 12 HbA1c	HbA1c change	*p‐*value (paired T)
All patients (*n* = 2162)	6.08 ± 1.34	5.95 ± 1.07	−0.13 ± 0.92	< 0.001*
DM patients (*n* = 685)	7.32 ± 1.72	6.87 ± 1.34	−0.45 ± 1.49	< 0.001*
Non‐DM patients (*n* = 1477)	5.50 ± 0.43	5.51 ± 0.51	0.01 ± 0.39	0.189
DM patients with cirrhosis (*n* = 130)	7.39 ± 2.13	6.78 ± 1.36	−0.62 ± 1.76	< 0.001*
DM patients without cirrhosis (*n* = 555)	7.31 ± 1.61	6.90 ± 1.33	−0.41 ± 1.43	< 0.001*
Non‐DM patients with cirrhosis (*n* = 180)	5.26 ± 0.47	5.34 ± 0.52	0.09 ± 0.36	0.002*
Non‐DM patients without cirrhosis (*n* = 1297)	5.53 ± 0.41	5.54 ± 0.51	0.003 ± 0.39	0.791
DM patients with pretreatment HbA1c greater than or equal to baseline average^a^ (*n* = 457)	8.04 ± 1.66	7.27 ± 1.31	−0.78 ± 1.61	< 0.001*
DM patients with pretreatment HbA1c less than baseline average^a^ (*n* = 228)	5.87 ± 0.45	6.08 ± 0.99	0.21 ± 0.95	0.001*

^a^ Baseline average of HbA1c: 6.5%.

* *p* < 0.05.

In the DM group, HbA1c levels significantly decreased from 7.32% ± 1.72% at baseline to 6.87% ± 1.34% at SVR12 (*p* < 0.001), whereas no significant change in HbA1c levels was observed in the non‐DM group (5.50% ± 0.43% at baseline vs. 5.51% ± 0.51% at SVR, *p* = 0.189). The reduction in HbA1c levels was greater in the patients with DM with cirrhosis than in those with DM without cirrhosis (0.62% ± 1.76% vs. 0.41% ± 1.43%; Table 2). Moreover, in patients with DM who had pretreatment HbA1c levels greater than or equal to the baseline average (HbA1c: 6.5%), a significant reduction in HbA1c levels was observed (*p* < 0.001). By contrast, no significant reduction in HbA1c levels was observed among the patients with DM who had pretreatment HbA1c levels less than the baseline average (Table 2).

### 3.3 Improvements in HbA1c Levels After DAA Treatment Across Pretreatment HbA1c Level Categories

Categorical changes in HbA1c levels after DAA administration are presented in Table 3. Significant reductions in HbA1c levels were observed across different pretreatment level categories in patients with pre‐DM (*n* = 687) or DM (*n* = 464). Among patients with DM, 114 individuals (24.6%) who had baseline HbA1c levels exceeding 6.5 successfully reached an HbA1c level below 6.5 at SVR12. Among patients with pre‐DM, 168 (24.5%) individuals who initially had baseline HbA1c levels between 5.7 and 6.4 improved to HbA1c levels below 5.7 at SVR12. However, after SOF/VEL therapy, 111 (9.6%) of all patients exhibited increased HbA1c levels, and 460 (67%) patients with pre‐DM and 182 (39.2%) with DM exhibited no significant changes in HbA1c levels (Table 3).

**TABLE 3:** Changes in HbA1c levels after SOF/VEL treatment across pretreatment HbA1c level categories.

	M3^a^ HbA1c					*p*
	> 8.5 (*n* = 75)	7.6–8.5 (*n* = 84)	6.5–7.5 (*n* = 250)	5.7–6.4 (*n* = 556)	< 5.7 (*n* = 186)	
Baseline HbA1c						< 0.01
> 8.5 (*n* = 126)	42	39	21	18	6	
7.6–8.5 (*n* = 104)	22	18	56	7	1	
6.5–7.5 (*n* = 234)	7	23	122	71	11	
5.7–6.4 (*n* = 687)	4	4	51	460	168	

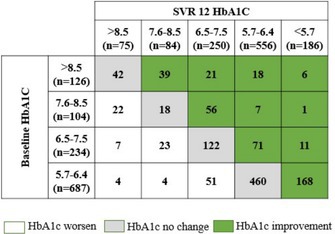

^a^ M3: 3 months after end of treatment.

**FIGURE 2:**
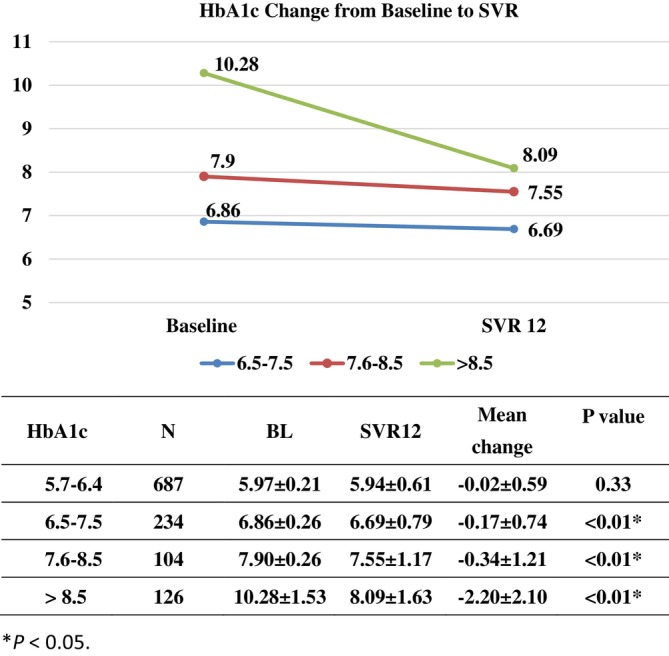
Line chart depicting the time‐course change in HbA1c levels versus the baseline in patients with DM. X‐axis, baseline, undetectable HCV RNA concentration at a 12‐week posttreatment follow‐up (SVR 12); Y‐axis, percentage with baseline A1c 6.5–7.5, A1c 7.6–8.5, or A1c > 8.5.

Significant reductions in HbA1c levels were observed among patients with DM across various pretreatment HbA1c categories. Specifically, the mean reductions in HbA1c levels were 0.17 ± 0.74 for patients with baseline HbA1c levels ranging from 6.5 to 7.5, 0.34 ± 1.21 for those with baseline HbA1c levels between 7.6 and 8.5, and 2.20 ± 2.10 for patients with baseline HbA1c levels > 8.5 (*p* < 0.01). However, among patients with pre‐DM (HbA1c levels between 5.7 and 6.4), no significant improvement in HbA1c levels was observed (0.02 ± 0.59, *p* = 0.33; Figure 2).

### 3.4 Factors Associated With Significant Reductions in HbA1c Levels

The magnitude of HbA1c level reductions varied among the studied subpopulations. Hence, we conducted a further analysis to identify the factors associated with significant HbA1c reductions in the cohort of 2162 HCV patients who achieved SVR. Patients who exhibited significant reductions in HbA1c levels had a higher age (62.1 vs. 60.6 years, *p* = 0.02) and higher BMI (25.5 vs. 24.5 kg/m^2^, *p* < 0.01). These patients also had an increased prevalence of hypertension (51.8% vs. 36.9%, *p* < 0.01) and exhibited elevated baseline levels of serum creatinine (1.11 vs. 0.99 mg/dL, *p* = 0.03), AST (64.1 vs. 54.5 U/L, *p* < 0.01), ALT (78.5 vs. 64.6 U/L, *p* < 0.01), triglycerides (123.3 vs. 106.3 mg/dL, *p* < 0.01), fasting glucose (136.2 vs. 107.1 mg/dL, *p* < 0.01), and HbA1c (7.1% vs. 5.7%, *p* < 0.01). Additionally, these individuals had a higher prevalence of DM (60.7% vs. 21.9%, *p* < 0.01), cirrhosis (17.6% vs. 13.2%, *p* = 0.01), and advanced chronic kidney disease (CKD; 4.5% vs. 2.4%, *p* < 0.01) than did the patients who did not exhibit significant HbA1c level reductions (Table 4). The results of a multivariate analysis indicated that fasting glucose levels (odds ratio [OR]/confidence interval [CI]: 1.01/1.00–1.01, *p* < 0.01) and DM (OR/CI: 3.73/2.73–5.10, *p* < 0.01) were significantly associated with HbA1c level reductions (Table 5).

**TABLE 4:** Univariate analysis of the factors associated with significant HbA1c level reductions^a^.

HbA1c significant improvement	Yes (*n* = 544)	No (*n* = 1618)	*p*
Age (years, mean (SD))	62.1 (13.0)	60.6 (12.9)	0.02
Male, *n* (%)	257 (47.2)	855 (52.8)	0.02
BMI (kg/m^2^, mean (SD))	25.5 (4.1)	24.5 (4.0)	< 0.01
Hypertension, *n* (%)	282 (51.8)	597 (36.9)	< 0.01
Creatinine (mg/dL, mean (SD))	1.11 (1.20)	0.99 (1.0)	0.03
eGFR (mL/min/1.73 m^2^)			< 0.01
> 90, *n* (%)	243 (44.8)	770 (47.6)	
60–89, *n* (%)	193 (35.6)	657 (40.7)	
30–59, *n* (%)	82 (15.1)	151 (9.3)	
15–29, *n* (%)	9 (1.7)	8 (0.5)	
< 15, *n* (%)	15 (2.8)	30 (1.9)	
AST (IU/L, mean (SD))	64.1 (58.9)	54.5 (46.5)	< 0.01
ALT (IU/L, mean (SD))	78.5 (79.8)	64.6 (70.1)	< 0.01
Platelet count (× 10^3^ u/L, mean (SD))	192.3 (69.6)	198.8 (72.4)	0.07
Triglycerides (mg/dL, mean (SD))	123.3 (145.0)	106.3 (58.9)	< 0.01
Fasting glucose (mg/dL, mean (SD))	136.2 (62.0)	107.1 (37.4)	< 0.01
HbA1c (%, mean (SD)))	7.1 (1.9)	5.7 (0.8)	< 0.01
Diabetes, *n* (%)	330 (60.7)	355 (21.9)	< 0.01
FIB‐4 (mean (SD))	3.0 (2.9)	2.7 (3.2)	0.08
Cirrhosis, *n* (%)	96 (17.6)	214 (13.2)	0.01
HCC history,*n* (%)	506 (93.0)	1,563 (96.6)	< 0.01
HCV RNA (log IU/mL, mean (SD))	5.9 (1.0)	5.8 (1.1)	0.05
HCV genotype 1, *n* (%)	189 (34.7)	642 (39.7)	0.04

Abbreviations: ALT, alanine aminotransferase; AST, aspartate aminotransferase; BMI, body mass index; FIB‐4, fibrosis‐4 index; HbA1c, glycated hemoglobin; HCV, hepatitis C virus; SD, standard deviation; SVR, sustained viral response.

^a^ A significant HbA1c level reduction was defined as > 75 percentiles of HbA1c reduction (0.2%) of the entire cohort after antiviral therapy.

**TABLE 5:** Factors associated with significant HbA1c level reductions.^a^

Predictors (mean (SD) or *n* (%)	HbA1c significant improvement	Unadjusted OR (95% CI)	*p* *	Adjusted OR (95% CI)	*p*
Age (years)		1.01 (1.00–1.02)	0.02	0.99 (0.98–1.01)	0.87
Gender					
Female	257 (23.1)	1		1	
Male	287 (27.3)	1.25 (1.03–1.52)	0.02	1.26 (0.98–1.62)	0.07
BMI (kg/m^2^)		1.05 (1.03–1.08)	< 0.01	1.01 (0.98–1.04)	0.59
Hypertension					
No	262 (20.4)	1		1	
Yes	282 (32.1)	1.84 (1.51–2.24)	< 0.01	1.33 (1.01–1.74)	0.04
Creatinine (mg/dL)		1.09 (1.01–1.18)	0.04	1.02 (0.90–1.15)	0.78
eGFR (mL/min/1.73 m^2^)		0.99 (0.99–1.00)	0.08	1.00 (0.99–1.01)	0.11
> 90	243 (24.0)				
60–89	193 (22.7)				
30–59	82 (35.2)				
15–29	9 (53.0)				
< 15	15 (33.3)				
AST (IU/L)		1.00 (1.00–1.01)	< 0.01	1.00 (0.99–1.01)	0.76
ALT (IU/L)		1.00 (1.00–1.00)	< 0.01	1.00 (0.99–1.00)	0.61
Platelet count (× 10^3^ u/L)		0.99 (0.99–1.00)	0.07	0.99 (0.99–1.00)	0.99
Triglycerides (mg/dL)		1.00 (1.00–1.00)	< 0.01	1.00 (0.99–1.00)	0.87
Fasting glucose (mg/dL)		1.01 (1.01–1.02)	< 0.01	1.01 (1.00–1.01)	< 0.01
Diabetes					
No	214 (14.5)	1		1	
Yes	330 (48.2)	5.49 (4.45–6.76)	< 0.01	3.73 (2.73–5.10)	< 0.01
FIB‐4		1.03 (0.99–1.06)	0.08	1.03 (0.97–1.11)	0.34
Cirrhosis					
No	448 (24.2)	1		1	
Yes	96 (31.0)	1.41 (1.08–1.83)	0.01	0.95 (0.64–1.41)	0.81
HCC history					
No	38 (40.9)	1		1	
Yes	506 (24.5)	2.13 (1.39–3.27)	< 0.01	1.23 (0.69–2.17)	0.48
HCV RNA(log IU/mL)		1.10 (0.99–1.21)	0.05	1.04 (0.93–1.17)	0.47
HCV genotype 1					
No	355 (26.7)	1		1	
Yes	189 (22.7)	0.81 (0.66‐0.99)	0.04	0.81 (0.63–1.04)	0.10

Abbreviations: ALT, alanine aminotransferase; AST, aspartate aminotransferase; BMI, body mass index; CI, confidence interval; FIB‐4, fibrosis‐4 index; HbA1c, glycated hemoglobin; HCV, hepatitis C virus; OR, odds ratio; SD, standard deviation.

^a^ A significant HbA1c level reduction was defined as > 75 percentiles of HbA1c reduction (0.2%) of the entire cohort after antiviral therapy.

* *p*‐values < 0.1 in the univariate model were included in the multivariate model.

## 4 Discussion

The findings of this study reveal a significant improvement in HbA1c levels after HCV eradication through SOF/VEL treatment among all patients who achieved SVR12. In patients with DM who achieved SVR, a significant reduction in HbA1c levels was observed across all subgroups. Furthermore, the reduction in HbA1c levels was more pronounced in patients with higher pretreatment HbA1c levels.

DM can be diagnosed using HbA1c levels, fasting plasma glucose levels, or oral glucose tolerance tests. HbA1c is particularly valuable for DM screening because of its high sensitivity and specificity [16, 17]. HCV infection increases the risk of pre‐DM and DM during the initiation of the insulin signaling pathway, comparing individuals with and without infection [23, 24]. Several factors predictive of DM development have been examined, including high BMI, male sex, and advanced liver fibrosis [25]. Liver cirrhosis can lead to hyperinsulinemia, which subsequently induces glucose intolerance [26]. Our previous study demonstrated that patients with high pretreatment glucose levels and liver cirrhosis may benefit more from HCV eradication in terms of reductions in 2‐h plasma glucose (2HPG) levels [7]. In the present study, HbA1c level reductions were more pronounced in patients with higher pretreatment HbA1c levels and in patients with DM and cirrhosis, consistent with the findings of another study [27]. Factors associated with significant reductions in HbA1c levels include high BMI and male sex, as reported in another study [25]. Furthermore, the results of a multivariate analysis revealed that the factors independently associated with significantly reduced HbA1c levels were fasting glucose levels (OR/95% CI: 1.01/1.00–1.01, *p* < 0.01) and DM (OR/CI: 3.70/2.70–5.06, *p* < 0.01).

Although glucose levels may be reduced following HCV eradication, the findings reported in the literature remain inconclusive [11, 12] because HCV infection is one of several risk factors for DM. These risk factors are complex and comprise both nonmodifiable factors, such as family history, age, ethnicity, and genetics, and modifiable factors, such as weight, physical inactivity, diet, high blood pressure, cholesterol levels, and smoking [28], which can exacerbate the symptoms of DM. In the present study, most patients with DM exhibited a significant reduction in HbA1c levels at SVR. However, 111 (9.6%) patients with pre‐DM or DM experienced increased HbA1c levels, and 460 (67%) patients with pre‐DM and 182 (39.2%) with DM exhibited no significant changes in HbA1c levels following SOF/VEL therapy. This variability may explain the inconsistent results in the literature.

Most studies have reported improvements in DM management either at the end of therapy or in the months immediately following treatment in patients with DM. A systematic review and meta‐analysis highlighted significant reductions in HbA1c and fasting plasma glucose levels after DAA treatment in patients with established DM [29]. However, Li et al. observed that although successful HCV treatment in patients with T2DM leads to a notable reduction in HbA1c levels shortly after therapy, these improvements are not maintained over the long term. Within less than 3 years of achieving SVR, HbA1c levels rebound to those similar to the levels of untreated patients or those for whom treatment failed, and some patients exhibit levels higher than those they exhibited before treatment [12]. Therefore, the sustainability of the reduction in HbA1c levels resulting from HCV eradication remains a matter of controversy. In consideration of this, large prospective cohort studies that incorporate thorough analyses of potential confounding factors—such as smoking status, steatosis severity, changes in BMI, physical activity levels, and adherence to antidiabetic therapy—are urgently required to evaluate the durability of these improvements.

In the present study, 99.2% of the patients achieved SVR12 after SOF/VEL treatment, which is consistent with the findings of an earlier study [15]. Additionally, in another study involving a different DAA regimen, patients with elevated pretreatment HbA1c levels appeared to experience more pronounced glycemic improvements after HCV eradication, aligning with the current findings [30]. Our prior nationwide investigations in Taiwan have also revealed that patients with CKD exhibited a greater likelihood of renal function recovery after HCV eradication with DAAs [8, 31]. In the cohort in the present study, 114 (24.6%) of 464 patients with DM who had baseline HbA1c levels of > 6.5% achieved posttreatment HbA1c levels of < 6.5% shortly after receiving DAA therapy, which is an ideal level consistent with the objective of diabetes control. Both successful HCV eradication and HbA1c level reductions can reduce the risk of DM‐related complications among individuals with T2DM, including progression to end‐stage renal disease, acute coronary syndrome, and retinopathy [32].

These findings underscore the hepatic and extrahepatic benefits of SOF/VEL treatment. Early eradication of HCV in patients with DM, particularly those with higher baseline HbA1c levels or cirrhosis, increases glycemic control and reduces the prevalence of extrahepatic complications associated with DM. Consequently, promptly identifying HCV‐positive patients with DM and initiating antiviral therapy to prevent fibrotic progression and reduce the risk of DM‐related extrahepatic complications is essential.

This study has several limitations, including its short observational period. However, we enrolled a large cohort of patients from multiple centers throughout Taiwan that reflected real‐world situations, and our findings demonstrate that even a slight reduction in HbA1c levels shortly after viral eradication has clinical significance. Additionally, because the data were derived from a registry rather than controlled clinical trials, we used a standardized database platform to mitigate reporting bias and confirm the results.

In conclusion, the elimination of HCV with SOF/VEL significantly reduced HbA1c levels, especially in patients with DM with higher pretreatment HbA1c levels and cirrhosis. The findings of this study reveal that treating HCV can alleviate both hepatic and extrahepatic symptoms, underscoring the importance of involving non‐hepatology specialists in HCV care.

## Conflicts of Interest

Ming‐Lung Yu: Research support (grant) from BMS, Gilead, Merck, and Roche Diagnostics; consultant of AbbVie, BMS, Gilead, Roche, and Roche Diagnostics; speaker of AbbVie, BMS, Eisai, Gilead, Roche, and Roche Diagnostics. Wan‐Long Chuang: Advisory board member for AbbVie, BMS, Gilead, PharmaEssentia, Roche, and Vaccitech; speaker for AbbVie, BMS, Gilead, and Roche. Chia‐Yen Dai/Chung‐Feng Huang: Speaker for AbbVie, BMS, Gilead, Merck, and Roche. Yi‐Hsiang Huang: Research grants from Gilead Sciences and AstraZeneca, honoraria from AstraZeneca, Gilead Sciences, Eisai, and Roche, advisory role for AstraZeneca, Eisai, and Roche. All other authors declare no conflicts of interest.

## Data Availability

The data that support the findings of this study are available on request from the corresponding author. The data are not publicly available due to privacy or ethical restrictions.
